# Challenges of design, implementation, acceptability, and potential for, biomedical technologies in the Peruvian Amazon

**DOI:** 10.1186/s12939-022-01773-7

**Published:** 2022-12-19

**Authors:** Tiana Bressan, Andrea Valdivia-Gago, Rosa M. Silvera-Ccallo, Alejandro Llanos-Cuentas, Daniel F. Condor, Pierre G. Padilla-Huamantinco, Stalin Vilcarromero, J. Jaime Miranda, Carol Zavaleta-Cortijo

**Affiliations:** 1 School of Engineering, University of Guelph, 50 Stone Rd E, Guelph, ON N1G 2W1 Canada; 2 Escuela de Nutrición, Universidad Peruana Cayetano Heredia, Lima, Peru Av. Honorio Delgado 430, 15102; 3 Unidad de Ciudadania Intercultural y Salud Indigena, Facultad de Salud Publica y Administracion, Universidad Peruana Cayetano Heredia, Lima, Peru Av. Honorio Delgado 430, 15102; 4 Instituto de Medicina Tropical Alexander von Humboldt, Lima, Peru Av. Honorio Delgado 430, 15102; 5 School of Nursing, Universidad Peruana Cayetano Heredia, Lima, Peru Av. Honorio Delgado 430, 15102; 6 Biomedical Informatics in Global Health Unit, Universidad Peruana Cayetano Heredia, Lima, Peru Av. Honorio Delgado 430, 15102; 7 CuidART-e Research Group, Universidad Peruana Cayetano Heredia, Lima, Peru Av. Honorio Delgado 430, 15102; 8 Health Innovation Lab, Institute of Tropical Medicine Alexander von Humboldt, Lima, Peru Av. Honorio Delgado 430, 15102; 9 Institute for Biological and Medical Engineering, Schools of Engineering, Medicine, and Biological Sciences, Pontificia Universidad Católica de Chile, Santiago, Chile Av Vicuña Mackenna 4860,; 10 Hospital Nacional Edgardo Rebagliati Martins (HNERM), EsSalud, Lima, Peru Av. Edgardo Rebagliati 490, 15072; 11 CRONICAS Centre of Excellence in Chronic Diseases, Universidad Peruana Cayetano Heredia, Lima, Peru Av. Honorio Delgado 430, 15102; 12 School of Medicine, Universidad Peruana Cayetano Heredia, Lima, Peru Av. Honorio Delgado 430, 15102; 13 The George Institute for Global Health, UNSW, Sydney, Australia; 14 Unidad de Ciudadanía Intercultural y Salud Indígena (UCISI), Universidad Peruana Cayetano Heredia, Lima, Peru Av. Honorio Delgado 430, 15102

**Keywords:** Biomedical engineering, Technology, Challenges, Healthcare access, Amazon, Peru

## Abstract

**Background:**

Biomedical technologies have the potential to be advantageous in remote communities. However, information about barriers faced by users of technology in general and in remote Indigenous communities is scarce. The purpose of this study was to characterize the leading challenges faced by researchers who have used biomedical technologies in the Peruvian Amazon.

**Methods:**

This exploratory, qualitative study with a phenomenological approach depicts the lived experience of participants who were researchers with experience working with biomedical technologies in the Peruvian Amazon in the past five years. Analysis was based on three core themes: design, implementation, and acceptability. Sub-themes included environment, community, and culture. Of the 24 potential participants identified and contacted, 14 agreed to participate, and 13 met inclusion criteria and completed semi-structured interviews. Results were sent to each participant with the opportunity to provide feedback and partake in a 30-minute validation meeting. Five participants consented to a follow-up meeting to validate the results and provide further understanding.

**Results:**

Participants recognized significant challenges, including technologies designed out-of-context, difficulty transporting the technologies through the Amazon, the impact of the physical environment (e.g., humidity, flooding), and limited existing infrastructure, such as electricity and appropriately trained health personnel. Participants also identified cultural factors, including the need to address past experiences with technology and health interventions, understand and appropriately communicate community benefits, and understand the effect of demographics (e.g., age, education) on the acceptance and uptake of technology. Complementary challenges, such as corruption in authority and waste disposal, and recommendations for technological and health interventions such as co-design were also identified.

**Conclusions:**

This study proposes that technological and health interventions without efforts to respect local cultures and health priorities, or understand and anticipate contextual challenges, will not meet its goal of improving access to healthcare in remote Amazon communities. Furthermore, the implications of corruption on health services, and improper waste disposal on the environment may lead to more detrimental health inequities.

**Supplementary Information:**

The online version contains supplementary material available at 10.1186/s12939-022-01773-7.

## Introduction

Biomedical technologies include equipment for prevention, diagnosis, and treatment of health conditions while involving the application of designing medical equipment to human testing (e.g., point-of-care testing) and conducting research (e.g., data collection and mobile phone diagnoses) [1, 2]. For remote Amazon communities, there is potential for technology to be of great use by increasing the accessibility of medical care from outside the community [3–5]. Biomedical technologies could be a solution to the limited access to healthcare services, specialists, and primary-care service providers, partially influenced by geographical distance. For example, capture and storage of digital images and medication adherence on mobile devices can be transmitted to a remote provider providing more frequent and accurate data to both researchers and physicians [6]. Despite the success and importance of digital health technologies, there continues to be challenges with use and implementation in rural settings across multiple countries and limited information for the Amazon.

Specifically, digital health technologies (e.g., health data (electronic records), telemedicine, mobile technology (mHealth)) are revolutionizing how healthcare is administered [7]. They have the potential to increase healthcare access and universal health coverage, decrease implementation costs, improve healthcare services and information, and successfully contribute to non-communicable disease prevention [8, 9]. In rural settings in Africa, it has been observed that digital health presents challenges resulting in slow technology adoption, such as a need for strengthened governance and legal frameworks, resilient health systems and communities, funding, and sustainable, long-term projects [8]. Other critical challenges for rural communities included poor infrastructure, unstable power supply and internet connectivity, unavailable maintenance, and technologies not adapted to the physical context [8, 10, 11]. Further challenges and factors will continue to rise with digital health technologies, such as improving interoperability to enhance communication and expand databases [12, 13] and the question of who will equitably fund digital technology in response to increased demand due to the increased accessibility and reduced cost [14]. These challenges with technology use could be echoed in other rural settings, such as the Amazon.

Regarding telemedicine, Bhaskar et al. (2020) analyzed the status of telemedicine and services pre- and during COVID-19, outlining differences in regions that affect the implementation [15]. For example, Thailand has a shortage of physicians, but extensive internet penetration, whereas China has larger inequities between rural and urban areas [15]. Latin America faces challenges with healthcare provider access between rural and urban areas and requires increased adoption, public policy and infrastructure development, and stakeholder support [15]; however, there is limited information on first-hand experiences with these challenges. Telemedicine also requires internet access and digital literacy, introducing language, culture, and socioeconomic context barriers in remote settings [15].

In addition to physical and systemic challenges, researchers working in settings outside of South America reported that implementation of technology in remote locations, particularly among Indigenous people, did not consider the local cultural context [16, 17]. Elements of cultural safety and cultural competence have been deemed important in achieving equitable health care [18]. Moreover, the World Health Organization (WHO) estimated that 70% of medical equipment comes from the most developed nations and does not work when reaching the developing world [19]. The need for adapting to cultural context paired with a dependence on foreign import of technology may be a barrier to acceptability in numerous communities.

The involvement of the engineering profession in understanding and addressing global health challenges has traditionally been limited. Whereas global health remains a primary concern in public health, public health challenges have rarely been framed as technical questions [20]. The field of engineering, including rapidly advancing health-related technologies, can play this role and has the potential to address health inequities and advance health systems in multiple at-risk settings, such as remote communities in low- and middle-income countries [21]. Most recommendations, however, have been developed for agriculture and sanitation [22], while for health or medical interventions, they are still scarce, especially for the Amazon [23].

To better understand which technologies are in use and the challenges faced by researchers using them, this exploratory study aimed to characterize the challenges faced by researchers who have used biomedical technologies in the Peruvian Amazon. Findings are intended to raise awareness of potential concerns for future interventions in the Peruvian Amazon and similar contexts. This paper will outline the challenges with the design, implementation, and acceptability of biomedical technologies and conclude with recommendations to emphasize that interventions and implementation of technology should respect local cultures, consider the local environment, and align with local health priorities.

## Methods

### Study design

This exploratory qualitative study uses in-depth interviews with a phenomenological approach, an approach that emphasizes the subjective and lived aspects of an experience. This study depicts the lived experience of researchers who used health-related technologies in the Peruvian Amazon and faced challenges in the field first-hand, as there is currently not enough information addressing these geographical areas. A qualitative study approach was selected to provide a starting point for this discussion on addressing health inequities with technological solutions in rural settings in the Amazon. This overlap between public health and engineering is not widely investigated. In addition, we believe that complex topics such as culture and health can be described through life experiences.

This study was built upon the work of van der Zijpp et al. (2018), which suggested the following areas to understand challenges in the context of health care: design, implementation, and acceptability [24]. Van der Zijpp et al. (2018) outline frameworks to describe the phenomenon of why technology is not widely used and reference the human-centred design process [25], the Technology Acceptance Model (TAM) [26], and the Unified Theory of Acceptance and Use of Technology (UTAUT) [27].

In addition to the main three themes, questions about challenges explored environmental, community, and cultural aspects. These questions were based on the work of previous researchers implementing technology in the Amazon [28, 29] and concepts from the literature on technology implementation [30, 31] and working with remote and Indigenous communities [16, 32]. The primary and secondary themes and descriptions were selected a priori based on the literature provided in Supplementary Material Table 4.

### Sampling

A purposive sample of study participants was recruited from a list of individuals who have used biomedical technologies in the Peruvian Amazon and were recommended based on the experiences of one co-author (CZ-C) who worked in this region. Potential participants were contacted in February 2020 by the primary researcher (TB) via email and were provided information to participate, which included the purpose of the study, ethics approval information, and contact information for interview booking. In the email, the researchers were also asked for suggestions of other Peruvian researchers who have worked in the Amazon. Inclusion criteria included health researchers who have used biomedical technologies over the past five years in the Peruvian Amazon or individuals who have experience designing biomedical technologies for the Amazon region.

Of the 24 potential participants initially identified and contacted, 14 agreed to participate, and only 13 participants met the inclusion criteria. The 11 people excluded after being contacted included one who did not work in the Amazon region within the past five years, two who did not accept to participate because they felt they did not qualify for the study’s objectives, and eight who did not respond to the initial or follow-up email.

### Data collection

A semi-structured questionnaire (Supplementary Material: Interview Questions) was used that focused on the three main themes: design, implementation, and acceptability of technologies. The questionnaire was pre-tested for content and context by one Peruvian investigator (AVG). Interviews lasted between 30 to 90 minutes and were audiotaped. Interviews were conducted remotely via teleconference or phone call between March 2020 and April 2020. Four of 13 interviews were conducted in English and nine in Spanish. The Spanish interviews were translated to English before analysis and reviewed by a Peruvian investigator (AVG) for accuracy. All participants responded to all 14 questions within the interview questionnaire; therefore, no transcriptions were discarded. Interviews were manually transcribed verbatim. All organization or participant identifiers were removed, and audiotapes and transcriptions were numerically coded.

### Data analysis

Qualitative thematic analysis was conducted by the primary investigator (TB) and reviewed by a Peruvian investigator (AVG) and a supervising researcher (CZ-C). When conflict codification arose, the three researchers discussed opinions to come to a consensus on the final codification.

Thematic analyses included manifest and latent analyses. During the manifest phase, researchers identified the following demographic information from participants after de-identification, including sex, job position, research focus, regions where they worked, nativity to the Amazon, use or implementation of technologies in remote or Indigenous communities, and years of experience working in the Peruvian Amazon.

The iterative process of data revision allows for identifying new emergent themes related to “meanings,” “process,” and “definitions” handled by participants about relationships with the community, types of technologies, and challenges. This process helped identify more prominent themes and relevant concepts within the transcriptions (e.g., *corruption*). Finally, the researchers took note of the recommendations to improve the design and implementation of technologies within remote Amazon communities based on participants’ perspectives. Quotes were used extensively to provide a detailed description of the data.

Participants were invited for a complementary 30-minute follow-up interview to validate emerging themes in the results and prioritize the topics for the discussion. Five participants accepted and participated in validation interviews.

### Ethics

Written or oral consent was obtained from each participant. A consent form was prepared to discuss the extent of the interview and the purpose of the study. This study was approved by the Cayetano Heredia University ethics board and the University of Guelph ethics board.

This study was built upon an extensive research collaboration between Peruvian and International researchers through the Indigenous Health and Adaptation to Climate Change (IHACC) Project. The IHACC project is a multi-year, international, trans-disciplinary, community-based initiative working with Indigenous populations to examine the health effects of climate change and develop an evidentiary basis for adaptation.

### Positionality

The first author is a Canadian woman of Italian descent and a native English speaker who completed a research internship for approximately three months in Lima, Peru, in 2019, under the supervision of a Peruvian investigator (CZ-C). She has since continued working with the Peruvian IHACC team. She received a second funding opportunity to return to Peru in 2020; however, she completed work remotely with the team due to travel restrictions related to the COVID-19 pandemic.

### Background

#### Healthcare in the Peruvian Amazon

**Fig. 1:**
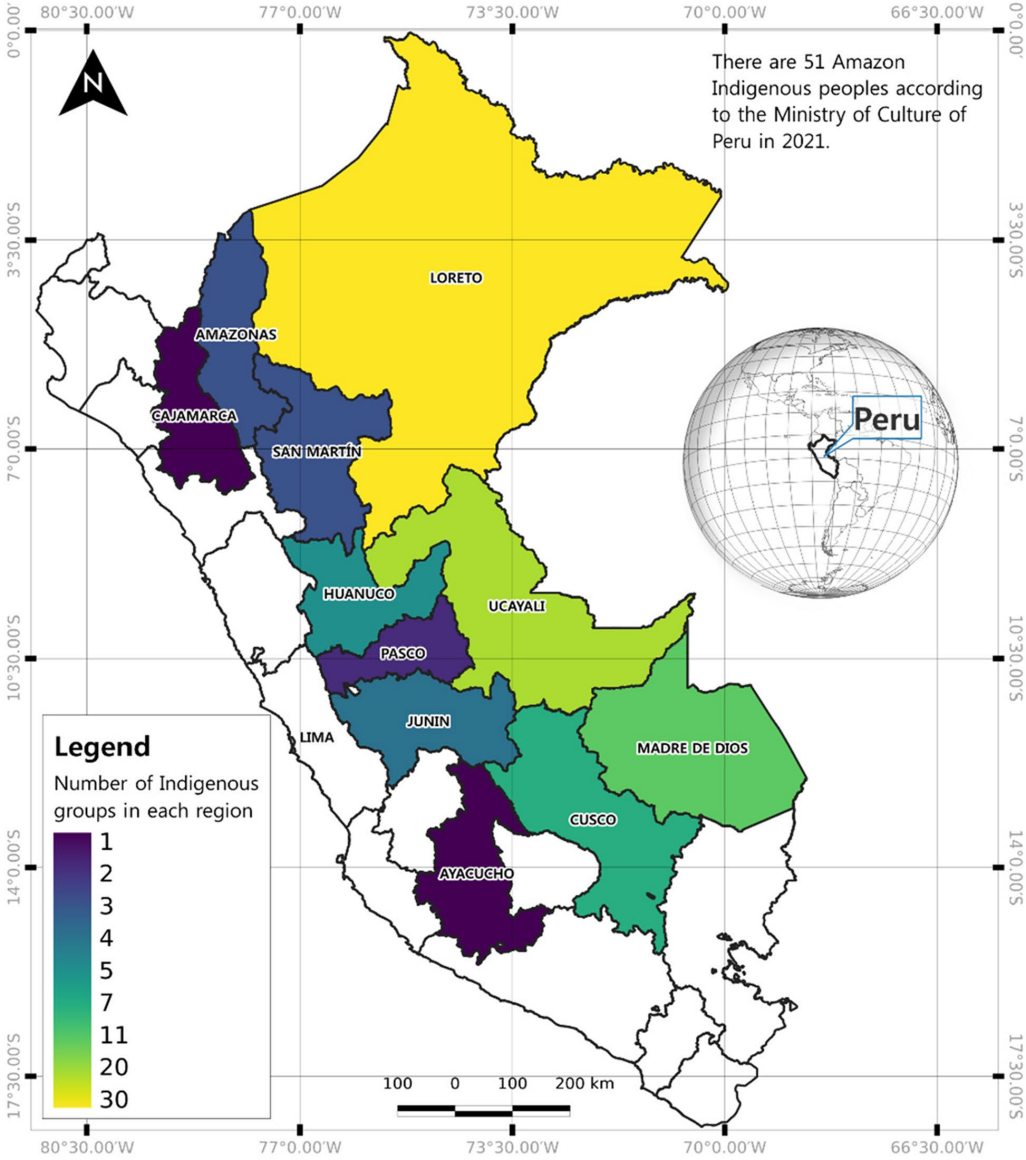
Peruvian map indicating regions where Amazon Indigenous populations are located

The Amazon region is a vast geographical and culturally diverse territory where formal health systems struggle to provide quality and timely health assistance [33]. Peru is home to 55 unique Indigenous populations, 51 of which reside in the Amazon and are predominantly localized in the following regions: Ucayali, Loreto, Madre de Dios, San Martín, Junín, and Amazonas [34]. Figure 1 depicts a map of Peru, showing the regions where Indigenous populations are located. A complete list of ethnicities and departments can be found in Supplementary Material Table 1.

These six regions contain two types of primary care institutions: health centres and health posts [5]. Health posts provide basic care and are a point of access to the healthcare system for rural populations [35]. Health posts are typically located in towns with less than 1000 inhabitants, minimal communication infrastructure (e.g., telephone lines), and challenges with transportation [5], partially due to difficult geographical access to healthcare facilities [35].

In contrast, health centres are often located in a provincial or district capital, have telephone lines installed, function under the direction of a physician, and are equipped to conduct diagnostic tests [5]. Several health posts depend on a single health centre; however, there is a need for better communication systems for physician consultation, surveillance, and supply ordering [5]. It was also reported that there was a lack of access to basic diagnostic tests for conditions like tuberculosis and malaria, or basic assessments, such as prenatal checks [36].

Primary care in Peru has been categorized by the Peruvian Ministry of Health based on available services from a Category I-1, including a health post with typically only a health professional, health technician, or both, to maximum I-4, which is a health center with hospitalization service [37]. This restructuring does not necessarily imply improved access to healthcare because geographical isolation and access to specialized care remain a challenge. The Andean and Amazon regions have fewer physicians and nurses than the national average. Loreto, the largest Amazon region, has 6.2 physicians and 8.8 nurses per 10,000 inhabitants, while Peru has 12.8 physicians and 14.1 nurses per 10 000 inhabitants [38]. Moreover, of 2,703 native communities registered in the 2017 census of native communities, 67% of these communities did not have health facilities [39]. Limited health services and human resources must be improved to close the health inequities gap in Peru, as a country, and more specifically, within the Amazon.

Facilities in most communities in the Amazon have registered community health workers (CHWs) [40]. CHWs, or health promoters, are native to the local community and are volunteer community members with basic training from the minister of health or other local, non-governmental health organizations in health promotion to help with campaigns. Studies have highlighted the role of CHWs in providing primary healthcare, as well as opportunities to improve their training [40, 41] and include them within the formal system with a salary [42]; however, these are only suggestions which have yet to be implemented. One Indigenous population in the Loreto and San Martín regions of the Amazon, Shawi, also known as Chayahuita or Kampo piyapi, currently faces health disparities due to only 13% of the communities having a primary care government health post and only 38% having a designated health promoter [43].

In addition, there are also local non-official health providers. In Spanish, they are called “*medicos,*” “*shamans,*” and “*curanderos,*” among other terms. A recent study found that more than half the people visiting an Amazon health facility sought health support from a Shaman or used medicinal plants to address their health conditions [36]. Traditional health beliefs are prevalent, and home remedies are preferred compared to professional healthcare in some Shawi communities due to a variety of factors such as accessibility, affordability, and perceived effectiveness [44–46]. Shawi perspectives on health tend to have a larger focus on emotional, collective, and environmental wellbeing rather than individual physical health, which is most often the focus in the biomedical definition of health [43].

#### Use of biomedical technologies in the Peruvian Amazon

Current biomedical technologies used in the Peruvian Amazon by researchers and health practitioners include, but are not limited to, stethoscopes, blood pressure monitors, hemoglobin testers and analyzers, and various other rapid diagnostic blood tests [47].

The Enlace Hispano-Americano de Salud (EHAS) is a foundation in Latin America that uses technology to improve healthcare in rural areas [48]. EHAS conducted a project in Peru between January 2001 and May 2002 to connect isolated health establishments and provide various services such as remote training and information exchange which had a positive impact on patients, health personnel, and the entire healthcare system [28]. Another successful project, Mamás del Río, is a maternal and neonatal health programme in the Peruvian Amazon where a volunteer CHW from each community participated in training to monitor pregnancy tests, develop a birth and emergency plan, and assess and monitor pregnancy with the use of electronic tablets [49].

Based on a variety of researchers’ experiences in Peru, it was concluded that mobile devices were accepted and effective for communicating public health messages and supporting diagnoses of infectious diseases [50–53]. However, most evaluations were performed in urban places such as Lima [37], a major urban centre. Moreover, it has been reported that Indigenous people are satisfied with telemedicine/telehealth but are “skeptical about its cultural safety,” raising questions about the acceptability of this service among communities [16]. Factors related to the “cultural competency” of healthcare providers and the type of condition being addressed have been highlighted as relevant for the acceptability of biomedical technologies [16, 32].

### Current frameworks for implementing biomedical technology

Various frameworks and resources exist and should be consulted to address challenges before designing health interventions with health-related technologies. Some of these frameworks include:

- Classification of Digital Health Interventions – WHO: provides a detailed digital health taxonomy and link to health system challenges, outlining how technology addresses specific health needs [54].
- Seventy-first World Health Assembly: outlined 11 key recommendations for member states, including an assessment of their use of digital technologies for health and how they can be integrated into current systems to identify priority areas [55].
- National eHealth Strategy Toolkit – WHO and the International Telecommunication Union (ITU): involves establishing an eHealth vision, developing an action plan, and monitoring and evaluation to recognize the potential for eHealth to improve health systems as a resource for governments [56].
- The Evidence Standards Framework for Digital Health Technologies: provides classification of technology and risk with best practices [57].

## Results

The majority of participants were male (9/13, 69%) and self-identified as health researchers. All participants had used biomedical technologies, and more than half of the participants used them among Indigenous communities, in the last five years. None of the participants identified as being native to the Amazon. Details on participant demographics are presented in Supplementary Material Table 2.

Technologies currently utilized as reported by participants are organized in Supplementary Material Table 3 and categorized into biomedical technologies for prevention, diagnosis, treatment, and general technologies. Examples include vaccines (prevention), hemoglobin analyzers (diagnostic), thermocoagulators (treatment), and drones for high-resolution images to understand pathologies related to spatial analysis (general).

### Overview

**Fig. 2:**
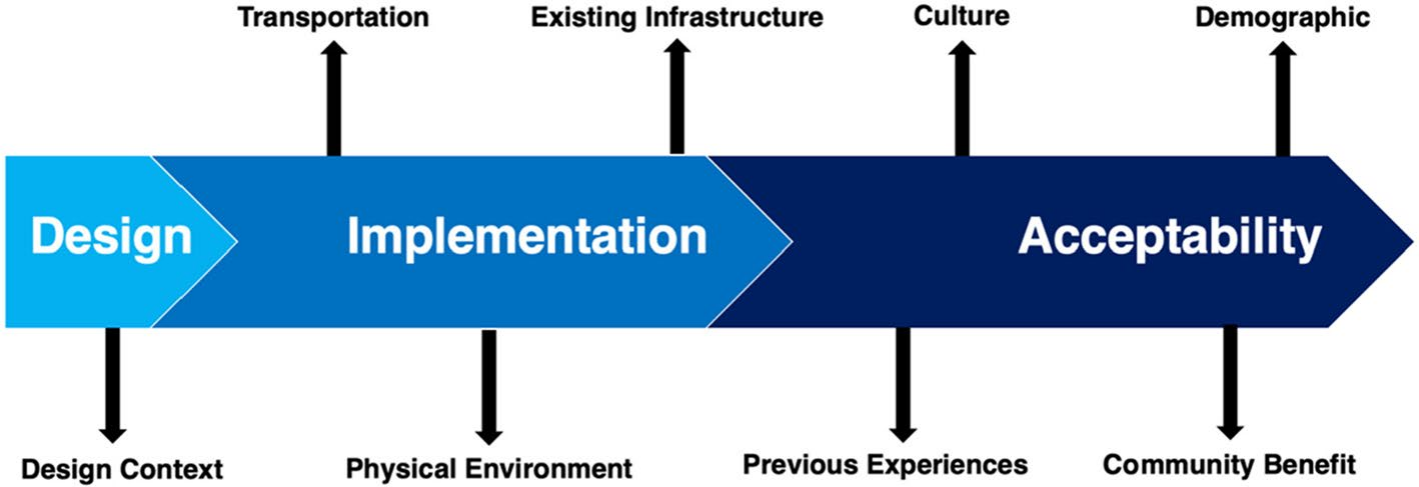
Depiction of the main challenges and barriers with technology, as described by participants

The main challenge presented in the *design* of technologies included the context in which the technologies were being designed. Concerning *implementation*, main challenges included transportation of technologies into remote Amazon communities, impact of the physical environment, and the absence or improper adaptation of technologies to benefit and adjust to existing infrastructure. Finally, *acceptability* of the community as perceived by participants, was affected by previous experiences with health interventions, cultural considerations, communication of community benefit, and differences in demographics including the effect of age and amount of formal education. Figure 2 shows the main challenges identified and described below.

### Design

#### Design context

Participants identified that technologies were not developed to be used specifically for the Amazon or remote Amazon communities. The majority of participants believed that producers or companies that develop technologies do not consider the local context when designing biomedical technologies.

Participants recognized two options for biomedical technology design. These include (a) paying for a technology on the market where it may be cheaper but not exactly what you want or (b) creating and developing your technology, which increases the cost, time, and maintenance of your interventions but is exactly what you need. Open-source hardware was also mentioned to be beneficial in knowledge sharing and multidisciplinary collaboration, as one can adapt and use a design to fit their context.

Concerns with technologies purchased on the market included that the technologies are mainly produced or designed in a North American or European context under different regulations. These technologies often do not work in Peru’s highlands or Amazon regions due to most design and testing being done in laboratory settings with highly controlled temperatures, electricity, and levels of biosecurity. In such, market technologies may have a higher failure rate, as noted by participants. For our participants, designing to fit the proper context was important and valuable to meet the community’s needs.

*“… if we opted for solutions that are on the market, for example, devices that already come ready to use or drones that only need to be purchased and taken to the field, the chance that this will fail is very high…” – (male, 0080)*

Additionally, it was mentioned, that market technologies are more expensive in these remote areas as there is less demand and, consequently, low production of local technologies. The increased costs of these technologies result from transportation to remote communities, import fees, and low-cost hardware from other countries with custom fees, delivery, and taxes. The low demand for technologies in these regions also results in companies raising fees to make a profit.

Participants recognized that technology failure or repair requires replacing equipment with components that cannot be found locally. Although there are companies that manufacture biomedical technologies, specific components and devices that meet certain requirements are not produced in South America as there are no local suppliers. This creates limitations with the maintenance and repair of equipment provided to communities and further reduces sustainability.

Participants also suggested that purchasing technologies on the market will limit the flexibility of the design. For example, manufacturer recommendations often have limited temperature ranges that do not align with Amazon temperatures which could be due to design or component requirements. Also, small engineering changes (e.g., colour) increase acceptance in communities, however, this is not always possible when devices are obtained from a standardized market purchase. A simple change such as colour could increase acceptability by adapting to personal preference and since various traditional beliefs associate different meanings to different colours. For example, colourful blood lancets improved children's acceptance and when technological devices were worn for a long period of time (e.g., GPS), women preferred being able to choose the colour. A participant also shared that one community felt that a white sheet in a hospital is synonymous with death and preferred light blue sheets.

*“People are not making good designs, they go with the mentality that they want to apply a design or information, but if they don’t know the community well or are not thinking about the benefit of them, it will very easily to make a mistake.” – (male, 0100)*

### Implementation

#### Transportation

Participants suggested that technology transportation introduced barriers that required extra preparation and time to complete their work when compared to working in urban areas or central cities. First, participants noted that travelling and accessing the communities may be difficult, as the distance to health centres and dispersed populations in remote communities require extra travelling time.

Concerning access to communities, multiple participants discussed that as distance increases from cities, access to some communities through the river, walking long distances, lack of bridges, or small trekking paths pose challenges when carrying or transporting equipment. It was also noted that it was important to transport equipment in the safest way, so that it is not damaged and does not promote deforestation or adverse effects on the local environment.

According to participants, transportation is also affected by seasons. In the dry season, access to communities was not always possible through the river as smaller rivers had little flow and were too dry to travel by boat. This required more time to travel by foot to each community and introduced additional difficulties in carrying equipment. In the wet season, walking paths become very wet (muddy) and slippery, presenting barriers and risks in transporting the equipment.

Carrying equipment made loading and maintaining various technologies complicated (e.g., a wooden stadiometer is very heavy and requires one person to carry it to each house in the community). Challenges of carrying equipment depend on the weight of the equipment, the distance to carry it, and the de-calibration of equipment during transportation. As mentioned by participants, technologies that were easy to transport included stethoscopes, blood pressure monitors, rapid diagnostic tests, and thermometers. Larger, more sophisticated equipment was more difficult and costly to transport.

*“There are several challenges. First, the equipment is used because it is [supposedly] lighter, if you go to the field, to the Amazon, those types of equipment doesn’t exist in the area and you have to carry them [all the way], it is a challenge because it also weighs, you have to load them and you also have to take care of them so that they do not fall, also, that they don’t have much movement because at all times they can de-calibrated…” – (female, 0080)*

#### Physical environment

The majority of participants described how biomedical technologies were unfit for the physical environment of the Amazon. Barriers to implementing technologies included elevated temperatures, high humidity, frequent flooding and rain, terrain, tree height, and available sunlight. Some participants discussed a percentage of equipment loss inherent to the fieldwork, specifically due to the lack of adaptability to the physical environment.

First, elevated temperatures and high humidity negatively affected the condition of equipment, reliability, and function. The adverse effects of environmental conditions included accelerated equipment deterioration and shortened lifespan, frequent de-calibration, and extended periods needed for cooling the equipment. However, only certain technologies were affected (e.g., hemoglobin analyzers) as some are more heat resistant (e.g., rapid diagnostic tests).

Flooding and rain were also common physical barriers due to their unpredictable and significant impact, as noted by participants. Flooding and torrential rain affected communication and electrical networks, resulting in power outages that prevented the constant flow of energy, presenting various risks (e.g., vaccine temperatures). Floods spoil much of the surrounding areas, amplifying humidity and deterioration of technological devices. Physical environmental barriers also amplify transportation challenges, as noted in the effect of seasons mentioned earlier.

Furthermore, uneven terrain posed limitations with current measurement technologies. For example, measuring accurate heights with a stadiometer was difficult as it was hard to find a flat area to measure on. Participants described that the terrain is also very rough and full of vegetation, challenging implementation of typical telecommunication networks or permanent infrastructure. Vegetation is also an amplifier of humidity.

High trees were discussed as a concern in disrupting antennas and limiting data transmission. Although solar technologies (e.g., solar microscopes) can help to overcome electricity barriers, one consideration was that they had to use an area in the community with direct sunlight, which involved being outside and moving around. Regarding sunlight, it was also challenging to view screens when working outside.

*“At that time [of the project], we did not have water-resistant equipment [cell phones], now we do, and we need [the equipment] to be more efficient with regard to battery use and perhaps with bigger and brighter screens, because when there is a lot of sun, the screens are not seen, but would require more power. We are at that point of finding that balance.” – (male, 0091)*

Other concerns included the biosecurity of blood samples and mosquitos. Since many samples are taken outdoors and not to a secured lab, drying blood samples attracted mosquitos.

#### Existing infrastructure

Participants discussed limited access to resources that supported the application of modern technologies within the communities, such as electricity and energy (e.g., single-use and rechargeable batteries), repair materials, basic communication infrastructure, and infrastructure in general. Additionally, they reported that they did not find appropriate capacity in some medical facilities to support the use of new technologies.

It was noted that there are different resource challenges in rural or dispersed populations when compared to urban centres. In the Amazon cities (e.g., Iquitos and Yurimaguas), there is a higher chance of 24-hour electricity access or laboratory access. In the field, when staying in rural communities for numerous days without returning to the city, there are significantly fewer resources, especially in Indigenous communities.

Many participants identified that the absence of consistent electricity in some remote communities does not favour the regular use of electricity-dependent technologies. Participants discussed using batteries as a power source to overcome this challenge. However, difficulties were presented with short battery life and lack of electricity to charge or recharge available batteries. For example, to understand the mobilization patterns of people, global positioning system (GPS)-like devices were worn on community members travelling for two weeks. The research team had no ability to recharge the batteries that only held a charge for approximately two days. This resulted in information loss, as no data was collected beyond the initial two days. Although batteries provided an adequate solution to the lack of access to electricity, participants were concerned as batteries sulfated quickly, and there was nowhere within the community to safely dispose of them. Solar panels were also used for energy; however, one limitation was that charging could only occur in direct sunlight during the day.

*“Sure, and that is why technologies should be used, at least with these point-of-care or devices that can help me without having to carry a huge device such as x-rays, which require electricity and a place, that is impossible or very difficult to install in a very remote area, this is exactly where this type of technology is needed; the idea is to make a field visit where it can be evaluated by different basic things to the populations residing there. It is necessary to develop this type of technology for this context, one that doesn’t need infrastructure.” – (female, 0088)*

Additionally, a prominent barrier was the need to access reliable communication infrastructure due to the limited antenna range in the region, as well as difficulty accessing internet connection and adequate bandwidth, which introduced challenges in data collection, speed of data transfer, and communicating with different remote areas.

A participant mentioned that in more remote areas, there was only radio transmission or private phones for communication, as compared to mobile phones which can be used in the city but have low connectivity in rural areas. Participants had difficulties with connectivity, bandwidth, weak signals, and constant interruptions in the electric power that prevented consistent signals from the telephone towers in remote communities.

*“When we implemented some projects there, the greatest difficulty was connectivity, the bandwidth was minimal, the signal was often very weak, there was constantly an interruption in the electric power that also prevented the signals from the telephone towers from ceasing to function, that for the infrastructure part.” – (male, 0093)*

On the subject of medical facilities and health posts in remote communities, it was noted that they have less of a capacity to provide services, as there are a limited number of trained healthcare personnel, supplied medicines, and infrastructure. For example, in one instance where participants were working in a health facility, they had enough microscopes but did not have enough trained technicians and health personnel to analyze or diagnose off the slides. Additionally, not only was there a need for internal or locally trained health personnel, but participants also noted the high turnover of out-of-region or foreign health professionals who worked in this region. The permanence of health human resources is limited and furthermore, there is little continuity of, trust in, and sustainability of projects.

*“Also, although they send doctors and nurses there, most of the doctors and nurses they send there, they don’t belong to the same area- they come from another place- usually from a big city. So, as you can imagine, if you come from a big city and you’re sent to the middle of nowhere- a place where it will take you ten days to get there by boat- you are not very keen to remain in your position too long.” – (female, 0033)*

Health services require more investment since much of the equipment was damaged and not repaired as there were few funding resources and minimal technical support for health facilities in the field.

### Acceptability of the community as perceived by participants

#### Previous experiences

A challenge perceived by participants was that community acceptability was impacted by negative or positive previous experiences and contact with health personnel, researchers, and technology. Participant perceptions included that distrust from the community towards health personnel from previous work that did not fulfil what was promised or breached trust may hinder cooperation or increase security risk. Participants also shared that positive previous experience and exposure to technologies increased collaboration and acceptance.

*“Regardless of technology, I have to mention, since we have not been the first group, and there have been groups before that may not left on good terms with the community, that may present a risk, since they probably promised things that they have not done… there were a couple of cases, where it happened that the previous group did not fulfil what was promised to the community, so it was a limitation, not so much on the part of technologies, despite not having electricity or electricity in the area.” – (male, 0091)*

#### Community benefit

Perceived community benefit was related to how the benefit of technology was communicated to the community and if the community agreed with such benefits. A challenge presented by participants was how to ensure that the community understood the benefits, direct impacts, and purpose of the technologies.

Participants suggested that the key to technology sustainability is having communities adopt it and make it their own. Participants brought to attention that although researchers think the benefit is practical, the community may have a differing perspective on what could benefit them. Participants suggested that community members who want to support these technologies can find solutions to mentioned barriers, such as inadequate connectivity and electricity, as they are more aware of specific points to find signals.

One example of a device with practical utility and community benefit included drones. The communities benefited as they could map their boundaries to distribute their territories, while researchers collected information about the land simultaneously. This occurred when researchers could clearly communicate the application or direct impact on health to community members. In contrast, an example of a challenge presented was with malaria interventions. There was a disconnect between understanding the benefit, as the community’s cultural belief of the cause of malaria was not related to mosquitos.

*“…but if they do not understand the beneficial part, it does not work.... Some will think that we are taking advantage of them. Communities will reject you if they feel they are being used, it depends on the way you enter with technologies… But if they see the benefit, they will accept you, and even more if you become friends with the community. They become more confident; they will ask you what the device does.” – (female, 0099)*

*“if the person that you involve in the study and you want him to be part of your support, if he really understands the benefit that he is going to have, his community and his family, and what he can do, he will become very engage, regardless of the limitations it may have, he will try to give the best it can, also to community agents, health promoters, people who already have these capacities or leadership skills, which are also very important when you are doing this, but the issue of communicating effectively, what the benefits are going to do, is a key factor so that it can be adopted and so that you can implement it properly and so that you can minimize the risks that may be associate each other, product of this context” – (male, 0097)*

#### Culture

Culture, reflected through language, community hierarchy, and kinships, was a prominent challenge for participants, since they were not native to the communities, and many did not speak the local language. Despite being Peruvians, participants recognized that they had to learn and respect the local culture. They suggested that working with native community members provided the insights needed to navigate the cultural differences.

Concerning language, not all community members spoke Spanish, and some members (e.g., women) spoke only their Indigenous language, creating limitations for participants when they wanted to introduce technologies and involve all community members. Often participants required a translator and despite challenges with communication, the interpretation, and back-translation, working with local translators who understood the community context and how to approach various groups was overall seen as beneficial. Some technologies, for example, the GPS, proved to be applied without a language barrier.

Second, participants mentioned community hierarchy and roles being important to understand and work with when implementing interventions and technologies. Specifically, the community leader (the *Apu*) must be approached first for community consent and negotiation to increase ease of entering the community.

Additionally, participants emphasized the value of understanding the kinships and relationships in the community, described as the social network, as a prominent factor to understand. Beyond working with the *Apu*, participants emphasized facilitating factors such as working with trusted community members that understand cultural differences and barriers. Some examples of working with trusted community members included projects involving the local health promoter or a local doctor, not originally from the region, but who stayed in the community for a long time and was well-respected and appreciated. Both instances increased the acceptance and collaboration of the community as perceived by participants. Understanding the social network also includes transmission of information verbally as the main form of communication and a focus on understanding and respecting familial roles and family systems.

*“when they learn, for example, girls learn with their mothers and boys learn with their fathers, the learning process to do thing in the community is a different process, because we learn from teachers, we have like a social institution that is the teacher and the school, and they don’t have this institution, they have elders, mothers, fathers, sisters, uncles, aunts, so they have all these institutions to teach, are part of the family relationships, so I think part of the culture, it’s that these different cultures pass through these different knowledge systems and educational systems. If where are going to teach them to use the technology properly, for example, to use vaccines, we can approach to the teacher at school but also, we can approach to the health post and also transmit information to more parts of the communities. […]the social network is important, but we need to identify how the social networks works in these different communities to try to send the message to them, the right message.” – (female, 0095)*

Multiple participants perceived technologies to be influencing traditional culture as there is a perceived shift away from maintaining culture and traditional and Indigenous medicines. However, participants described that it is a choice and right to globalize, but the consequence or risk as perceived by participants, is that traditional Indigenous cultural practices could disappear faster.

*“…they have easy access to cultural features of western or far eastern culture, it has quite an influence, it is changing their values. The risk for me is this, young people are seeing what is valued in other places, the music, the clothing, the activities they carry out and that can make them lose their values due to the local culture, it is observed in many places that young people do not want to dress as they traditionally dressed, the members of the communities do not appreciate the elderly and end up abandoning their communities, …, and going to larger cities, this makes the communities reduced or almost to the point of disappearing because all the young people left. This comes before the introduction of technologies [and technologies have made it easier] …Whether it is good or bad is debatable, but it is what is happening … […] This process is happening, you could say they have every right to globalize, to yield to international culture, and they are right and they have individual rights, but the consequence is that culture will disappear in a generation.” – (male, 0101)*

Additionally, participants perceived that traditionally and still today, most community members will seek a local shaman or curandero (member in the community that knows about the plants and natural medicines) before seeking treatment or medical assistance from Western medicines. Participants perceived that this is changing, as there is increased access and interest in Western medicine to complement their traditional systems.

*“it was not a population that only depended on traditional medicine, that had already changed, they wanted medicine or pills, or something that will help them solve that specific health problem, even, if they told us about other respiratory diseases, they already used medicines, they had a medicine cabinet, this population, as there was oil extraction, the company facilitated the installation of the medicine cabinets, and they regularly supplied them with drugs and they knew and had access, there was an interest in accessing more” – (female, 0088)*

#### Demographic

The majority of participants described their perception on the effect of demographic features (e.g., age, gender, education level) on technology acceptability. Regarding age, the majority of participants perceived the younger generation to be more accepting, curious, and open to using new technologies. Participants described that those who have had access to technologies might be more receptive and learn quickly. Concerning gender, some participants suggested that women are more accepting of technologies and are open to sharing feedback. As described by participants, other accepting groups included mothers interested in improving their children’s health and schoolteachers who were open to sharing technologies with students. The least accepting, as perceived by participants, included the older population. Others, more specifically, included traditional healers.

*“…depends on the age of the people, older people don’t take risks and they don’t accept it. On the other hand, the young people who had left the community and return with new technology, they do accept it easily, depends on how much access they have had when they migrate or left … everyone wants to learn, especially when you have a young population… there are groups, especially healers who are very reticent, they have traditional knowledge that they do not easily change, women are more collaborative.” – (male, 0100)*

Participants also perceived education as a factor affecting the acceptability of technologies. Barriers perceived did not include the willingness to learn or receptiveness, but rather the different focus on knowledge systems such as familial systems (e.g., daughters learning from mothers, learning from elders, uncles, aunts, etc.). Participants described that many community members and CHWs did not practice reading or writing Spanish, which introduces literacy challenges because directions and consent are often provided in written format, mainly in Spanish.

#### Impacts of technology among Amazon communities

To align with the perceptions of our participants, impacts were not divided into positive and negative impacts, and instead focused broadly on concerns about the impact of technology.

As perceived by participants, technology has improved and increased access to information and speed of results for both community members and researchers. Some advantages with increased access included the ability to collect and store more data remotely and efficiently, assisting in data collection and analysis. Collecting more data can be beneficial to inform decision-makers.

Participants also shared that with more prevalent chronic diseases (e.g., diabetes, hypertension) being discovered in these communities, it is beneficial to have access to modern technologies that have proven useful in diagnosing and monitoring these conditions. Participants also mentioned that biomedical technologies and technologies in general can provide solutions to healthcare gaps in remote areas. This includes exposure to new information and faster methods of communication for preventive actions and treatment.

Participants mentioned that with increased access and exposure to foreign information, which has been accelerated by technologies (e.g., cell phones), there is an increased risk of exposure to “fake news” and misinformation, which can influence fears of technology and challenge community knowledge and beliefs. Individuals’ beliefs and values can be impacted without proper training in critical analysis of information and technology-use.

### Complementary challenges

Participants discussed complementary challenges that occurred in parallel to the main themes identified. Participants described various incidents related to (a) corruption in authority, (b) waste disposal, (c) technology theft, (d) cost, (e) project timeline, and (f) invasiveness of technology.

#### Challenges with “corruption in authority”

Participants discussed the “corruption in authority” as a challenge. As reported by a participant, corruption in authority amplifies challenges, as there is often a focus on profit or political gain as opposed to public benefit.

*“…the challenge is for political decision-makers to understand it [implemented technology], even more complicated, because there is a high level of corruption and the first thing, they evaluated is to see how they will benefit from it. If they do not clearly see an economic personal benefit, they do not support, that is another problem…” – (male, 0100)*

Concerns were expressed with the sustainability of projects that are not priorities of the regional governments due to “corruption in authority”, and that authorities need to recognize that they cannot assume that a standard solution will work for everyone.

Participants shared concerns about excluding local priorities in decision-making and lack of support to maintain projects implemented by authorities. For example, one participant discussed frustration with government investment in a large project that was initiated but not financially maintained, resulting in technology deterioration. Concerning authority in general, participants shared that there is often slow decision-making and a slow adoption of technologies on this level.

#### Waste disposal

The technologies used by the participants within the communities were often non-recyclable, and participants perceived a need for recycling support. This resulted in contamination and improper disposal of equipment. For example, a reported concern was that there was no place to properly recycle batteries which are used frequently, and concerning equipment in general, it was reported that the community often disposed of it into the environment, unaware that they were contaminating it. Another participant reported that there might not be a clear distinction between garbage types as they throw away unused technologies (e.g., old radios, flashlights with batteries) into the river.

*“But one of the aspects that has left me worried, that unfortunately, due to the scope of the project we could not evaluate or design, would be to work on the environmental impact that the implementation of these devices can do and how it is waste management, I think it is something that is not done correctly in our country, is not done correctly in the city, it would be a much worse situation in these communities. The management of waste and the environmental impact that the application of technologies could have, in this case more in terms of hardware, would be once as well as once they are used and meet their life cycle, how waste is managed, that is something that would be an effect, one of the most important and strongest that these ecosystems could have. ... I think that using tools or technologies that end up causing damage to the environment itself and to the population, and on the other hand the management of waste that later generates an environmental impact and that is not in a sustainable way” – (male, 0097)*

#### Technology theft

Some participants were concerned with technology security, as some participants have been affected by theft. For example, it was reported that easy-to-use, portable technologies such as tablets, phones, or cameras introduced some risk, as individuals who were interested and curious may take the technologies for themselves. Improving safety when using tablets involved techniques to avoid visibility of the devices. In two situations where a camera was stolen, one was not found however, the other was retrieved once addressing the concern with the community authority.

*“Finally, security, you have to see how safe the site is, no matter how far away communities we find, many times there will be people who will want to steal the equipment … It has a lot of influence, if we do not have security within the community, you leave a team and after a week it was lost, you already lost an opportunity to continue collecting data,” – (male, 0093)*

#### Cost

It was reported by multiple participants that cost can impact technology. For example, more sophisticated or better-quality technologies, as well as continual maintenance, is generally more expensive. Batteries, transportation, energy, and energy storage increase the cost of projects and equipment. This can occur during design, implementation, and acceptability, as there are budget limitations to develop and maintain technologies. For example, in one report of using a standard GPS unit, there were many challenges until purchasing a more costly but higher quality GPS that was weatherproof, sturdy, and had better resolution.

#### Project timeline

Many participants also described the demand for, or anticipation of, extra time required to complete certain tasks when compared to working in urban settings. For example, regarding the humidity, it was reported that a server was running very slowly and sometimes stopped functioning. The server was not able to be used for multiple days, hindering the implementation and project timeline. Additionally, many participants reported that transportation between small communities takes time by boat through the river and that this must be accounted for. Furthermore, ample time must be considered for consent, training, and negotiation. For example, a consultation with an Indigenous leader that was planned to happen in one afternoon lasted five days to reach an agreement. This was because the community also had specific requests such as including an Indigenous health technician, local workers, jobs, and opportunities to train their people for future experiences.

*“I think, we as a western scientist and developers of technology, we think that everything is going to be talked in one meeting or two meetings, but with the communities their culture is to involve all the people, try to spend time with the community, they have different values, different ways of learning,” – (female, 0095)*

#### Invasiveness and perception of fear

Many reported that increased complexity or medical invasiveness (e.g., puncturing the skin) of testing and technology required more explanation and discussion for communities to accept it. This was mentioned as a potential barrier to acceptability of the community. For example, a tensiometer resulted in perceived fear from the applied pressure, which was resolved through an explanation of the process. A second example involved using colourful hemoglobin analyzer lancets that contained small needles. Both adults and children accepted the colourful lancets, since they only required a small drop of blood in contrast to a venipuncture that collects more blood.

*“For example, with the rapid [diagnostic] test … they were very happy with that. But I mean, the people likes that, you know, it’s because, less invasive. It's a test that's more accepted … if your test is invasive somethings going to be hard for some people. But for example, to use some tests that only need a puncture in the skin for example, in your hands and a couple of bloods- that's perfect. It works very well, people know, and are well adapted to that. But when you need to take a sample from their arms, … The complexity is different” – (male, 0098)*

### Recommendations from participants

Recommendations mentioned by participants are provided below in Table 1.

**Table 1:** Participant recommendations for technology design, implementation, and acceptability

**Design**	
• **Participatory design, co-design, and community-based approaches can enhance acceptability.** o Working with the community to develop and adapt technologies has potential to improve engagement and increase likelihood of communicating and discussing community benefit.	
**Implementation**	
• **Desiccants, coolers, and dehumidifiers for humidity and water-resistant technologies can help to overcome environmental challenges.** • **Consideration of where technologies will be stored and how they will be cleaned is important to prevent insect nesting and contamination.**	
**Acceptability**	
• **Working with people who understand local customs, roles, and community dynamic (e.g., CHWs, local translators, community authorities, anthropologists) can improve cultural understanding.** o Working with people who understand the local context could assist with developing an understanding of how to appropriately involve community members. Each community is different and standardized solutions are impractical. • **Address literacy and language barriers, and communicative strategies with visual materials (e.g., Rotofolios and pictures).** o Communication materials included figures or drawings with verbal explanations, instead of written words alone. Pictures or videos that are familiar to the local context or adapted to their context, rather than from urban areas or cities, is a useful strategy for health personnel. • **Committing to use the information collected with technology can enhance community benefit and acceptability.** o There should be a clear commitment on who will use the information gathered, how it will be used, and how the public or community will benefit. Information administration should also consider social impact of results such as information spread on sensitive topics. • **Supporting a salary for CHWs or health promotors.** o Given their time and contribution to community health, participants suggested providing health promotors with an official salary, so that they can support their families financially.	

## Discussion

This study addresses the gap in the literature on biomedical technologies that are used to improve access to healthcare and conduct research in remote communities, specifically in the Amazon, by identifying challenges and recommendations for technology implementation. This exploratory study uniquely captures the first-hand experiences and perceptions of the design, implementation, and acceptability of technologies in Amazon communities from the point of view of health researchers, designers, and implementers of technology who have worked in the Amazon. This study also characterizes context-specific challenges that hinder the uptake of technologies in these areas and populations that have historically received limited attention in the literature, particularly in technological and health fields.

The majority of the technologies discussed by participants involved diagnostic devices, with the least discussion around treatment and little discussion on prevention. These findings could be due to the limited sample size of the study or could be an overlooked factor influencing health inequalities. Minimal discussion on treatment may also align with what the researchers perceived community concern to be around being socially accountable and not only collecting data but using it to motivate short- and long-term change.

Mobile health (mHealth) was one preventative technology mentioned by participants as a successful tool to monitor pregnancies. Various projects have also utilized mHealth to increase access to health services that are difficult to transport to rural communities, such as mental health support in Latin America [28] and client education with Indigenous populations worldwide [16]. Mamás del Río, a mHealth project in the Amazon, had reported rapid communication and continued data collection and monitoring, especially during the COVID-19 pandemic [49], emphasizing the benefits of strengthening community capacity and working with CHWs to build resilient communities and health systems. Capacity building and working with CHWs were also recommended by participants in this study. This study found that limitations to consider when implementing mHealth and digital health technologies include literacy and digital capacity to use these devices, which agrees with the literature on lessons learned in mHeath for public health in Peru [47].

Furthermore, demographic factors such as age were mentioned by participants as a factor affecting acceptability, noting that the younger population that travelled into the city was perceived to be more open to technology. Similarly, a cross-country analysis in Europe acknowledged that younger populations, higher education, employment, and proximity to an urban environment contributed to higher digital skill [58]. This correlation between income and internet access and digital skill development should raise concern that if such disparities occur within a high-income region on a national scale, potential drastic inequities may exist on a global scale, such as within remote and rural settings in the Amazon.

The majority of participants perceived that technology developers were not always designing within or for the Amazon context, which presented challenges when purchasing technologies for use. This perception aligns with previous reports that indicate that local technology development is scarce in Latin America [19] and that this region “is highly dependent on imports of medical products as less than 4% of these are sourced within the region itself” ([59], p.1). Moreover, imported equipment in Latin America is often of poor quality, with 96% failing after five years post-donation and 39% never working due to a lack of training manuals or accessories [60]. This dependence on foreign technologies has made Latin America more vulnerable during health crises, as observed throughout the COVID-19 pandemic, and increased challenges in providing healthcare services to communities [59, 61]. A potential solution provided by participants in this study to improve the context-specific design and increase access to medical equipment within Latin America is open-source hardware which has also been proposed in analyses of open-source hardware for medical devices [62].

Findings throughout this study reinforce the importance of cultural safety and cultural competence among those working with biomedical technologies, from engineers designing technologies to health researchers and technology implementers in settings like the Peruvian Amazon. Participants mentioned that concepts around cultural safety and training to communicate technical information with communities are often left out of mainstream education yet are essential for developing and implementing culturally appropriate health interventions. This aligns with previous studies outlining cultural safety as a key competency for healthcare providers and workers that work with Indigenous peoples [18].

Complementary challenges reported in this study, such as *corruption,* revealed the complexity of implementing biomedical technologies and achieving a sustainable healthcare system within the Peruvian Amazon. *Corruption* in this context may be defined as “abuse of entrusted power for private gain” [63]. For example, corruption has an effect on healthcare systems and cancer care in Africa [64] and has been discussed as a large global health challenge [65]. Good governance is absent in many African countries [64], and consequently, there is inadequate prioritization of projects; public health budgets and funding are used for private gains; services are provided impractically to sway elections; and the quality of medical care has, as a result, deteriorated [64]. Similarly, corruption in Peru is widespread, which has also impacted institutional trust and political engagement [66]. Digital tools may be useful in increasing transparency and anti-corruption in medicines e-procurement, but adoption of these systems remains slow in even highly developed countries [67].

Another concern raised by participants was that technologies were disposed of into the river and natural, local environment due to inadequate waste disposal. Various studies conducted in the Peruvian Amazon have suggested that the discharge of lead by manufacturing, use, and disposal into the environment could be a potential source of lead blood contamination among Indigenous children [68, 69]. This poses a significant environmental and health risk for communities. One way to prevent, mitigate, or overcome this challenge would be to measure the environmental impact of interventions and, more specifically, complete a cradle-to-grave or cradle-to-cradle life cycle assessment to assess all stages of a product’s life from raw material extraction to disposal.

Another complementary challenge reflected throughout design, implementation, acceptability, and proposed recommendations included cost and limitations in funding. Remoteness, travel time, equipment importing, and negotiation time increased the cost of interventions. Another challenge included the influential role of funders in shaping research since non-traditional approaches, such as co-production, a recommendation by participants, and environmental protection, often do not fit traditional health funding protocols. A previous study indicated that co-production requires more time and resources to build a horizontal power base, trust, meaningful partnerships, and sustain activities [70]. However, co-design and co-production often have more successful innovations and better cooperation between people involved, potentially reducing development costs and time and improving user satisfaction while building long-term community capacity [71]. Tools such as equipment, medicines, and data are foundational to high-quality health systems but require attitudes, skills, and a willingness to learn within and outside the community to benefit the people it serves [72].

In the case of the Peruvian Amazon, working with CHWs, as recommended by participants in this study and proven successful in a previous study [49], is an opportunity to navigate local culture through co-design and implementation of biomedical technologies. Moreover, issues related to the long-term health of the physical environment (e.g., waste disposal), as emerged in this study, require consideration when developing protocols for technology use. A shift is needed from short-term partnerships and funder or policy-driven agendas toward long-term, meaningful partnerships that prioritize community needs and sustainability [70].

Factors that influence the acceptability of digital health by stakeholders, such as confidentiality of medical information, problems with the design of the device, language, and policy, are outlined in detail in the WHO Guideline Recommendations on Digital Interventions for Health System Strengthening [14] and align with the challenges presented in this study. The WHO Guideline outlines an evaluation of digital health initiatives and includes considerations for acceptability and feasibility of different stakeholders, recommendations to overcome challenges, and implementation considerations [54].

Furthermore, considerations and best practices for scaling digital health initiatives in low- and middle-income countries that may address challenges described in this study and are derived from successful case studies in Ghana, Africa, include (a) programme characteristics (e.g., user-centered design), (b) human factors (e.g., training and motivation of end-users), (c) technical factors (e.g., simple versus complex technologies), (d) the healthcare ecosystem (e.g., financial support), and (e) the extrinsic ecosystem (e.g., available electricity, hardware) [73]. There are also various working groups and advisory boards in South Africa, India, Rwanda, and Uganda that advocate for improved environments for digital health [73].

There are few resources that outline challenges faced, recommendations, and lived experience for those working or who wish to work with technologies in these regions. This study aims to raise awareness of the importance of local context in designing and implementing health technologies in rural and remote Amazon communities. It is critical to recognize that such interactions require investing time and energy in appropriate and respectful engagement with Indigenous communities and the prioritization of adequate context-sensitive solutions, including the long-term associated responsibilities such as training of human resources and waste disposal.

### Limitations

Limitations in this study included the limited sample size and purposive selection of participants, which may impact the generalizability of the study findings as they may not reflect the experiences of all researchers that have worked in the Amazon or designers of technology. To minimize limitations, strict inclusion criteria was used, inviting participants with experience working in the Amazon, in recent years.

Given the scoping nature of the study, another limitation is the subjectivity of some themes, such as acceptability for the community, which could be overcome in future studies by reaching out to local community members directly impacted by the technology. All participants have worked in the Amazon; however, none were local community members or those who were impacted by use of the technology. Eliciting the challenges and preferences, including acceptability, of Indigenous communities with technologies would be ideal; however, not all technologies are ubiquitous, hence such approach was deemed logistically challenging given the wide range of health-related technologies available from basic malaria rapid diagnostic testing to drones and cell phones and the few Indigenous communities that have experienced them. In this way, the study is more heavily weighted on design and implementation.

An additional limitation includes that the interviews took place remotely, due to restrictions in place during the COVID-19 pandemic and may have limited further analyses by direct field observation.

## Conclusion

This study outlined technologies used for prevention, diagnosis, and treatment and successfully characterized challenges with the design, implementation, and acceptability of technologies in the Amazon through the perspectives of health professionals who have worked in these regions. Gaps in the literature regarding contextual design, cultural safety, and recommendations specific to the Amazon were addressed. Findings indicated that a focus on diagnosis rather than prevention or treatment, foreign import of technology, corruption of authorities, improper local waste disposal, and limitations in funding could all pose barriers to technology use for equitable health care in the Amazon. Addressing challenges posed by health inequities with technological solutions must begin to include transdisciplinary collaboration between engineers and the public health sector, health researchers, and communities. This collaboration can maximize the potential of these technologies and ensure interventions are culturally and environmentally appropriate.

## Supplementary Information

**Additional file 1. ** Supplementary Material [74, 75].

## Data Availability

Audio recordings and transcripts analyzed in this study are not publicly available as adequate deidentification is not possible to maintain confidentiality. However, deidentified information is available from the corresponding author upon reasonable request.

## Abbreviations

TAM: Technology Acceptance Model
UTAUT: Unified Theory of Acceptance and Use of Technology
IHACC: Indigenous Health and Adaptation to Climate Change
GPS: Global Positioning System
CHWs: Community Health Workers
WHO: World Health Organization
mHealth: Mobile health
ITU: International Telecommunication Union
