# Supplementary material for: Challenges of design, implementation, acceptability, and potential for, biomedical technologies in the Peruvian Amazon
Source: Int J Equity Health. 2022 Dec 19;21:183. doi: 10.1186/s12939-022-01773-7 (PMC9762865; doi:10.1186/s12939-022-01773-7)
Supplement: Supplementary file 1 — Additional file 1. Supplementary Material [74, 75]. [file 12939_2022_1773_MOESM1_ESM.docx]

# Supplementary Material

### Table 1 Complete list of 51 ethnicities within the Peruvian Amazon and department where they are located (74).

| **Indigenous Ethnicity** | **Region where they are located** |
| --- | --- |
| Achuar | Loreto |
| Amahuaca | Madre de Dios, Ucayali |
| Arabela | Loreto |
| Ashaninka | Ayacucho, Cusco, Huánuco, Junín, Loreto, Madre de Dios, Pasco, Ucayali |
| Asheninka | Loreto, Pasco, Ucayali |
| Awajún | Amazonas, Cajamarca, Loreto, San Martín, Ucayali |
| Bora | Loreto |
| Cashinahua | Ucayali |
| Chamicuro | Loreto |
| Chapra | Loreto |
| Chitonahua | Ucayali |
| Ese Eja | Madre de Dios |
| Harakbut | Cusco, Madre de Dios |
| Ikitu | Loreto |
| Iñapari | Madre de Dios |
| Iskonawa | Ucayali |
| Jíbaro | Loreto |
| Kakataibo | Huánuco, Ucayali |
| Kakinte | Cusco, Junín |
| Kandozi | Loreto |
| Kapanawa | Loreto |
| Kichwa | Cusco, Huánuco, Loreto, Madre de Dios, San Martín, Ucayali |
| Kukama Kukamiria | Loreto, Ucayali |
| Madija | Ucayali |
| Maijuna | Loreto |
| Marinahua | Ucayali |
| Mashco Piro | Madre de Dios, Ucayali |
| Mastanahua | Ucayali |
| Matsés | Loreto |
| Matsigenka | Cusco, Madre de Dios, Ucayali |
| Muniche | Loreto |
| Murui-Muinanɨ | Loreto |
| Nahua | Ucayali |
| Nanti | Cusco |
| Nomatsigenga | Junín |
| Ocaina | Loreto |
| Omagua | Loreto |
| Resígaro | Loreto |
| Secoya | Loreto |
| Sharanahua | Ucayali |
| Shawi | Loreto, San Martin |
| Shipibo-Konibo | Huánuco, Loreto, Madre de Dios, Ucayali |
| Shiwilu | Loreto |
| Ticuna | Loreto |
| Urarina | Loreto |
| Vacacocha | Loreto |
| Wampis | Amazonas, Loreto |
| Yagua | Loreto |
| Yaminahua | San Martin, Ucayali |
| Yanesha | Huánuco, Junín, Pasco |
| Yine | Cusco, Loreto, Madre de Dios, Ucayali |

### Table 2 Participant descriptions and demographics including profession, research focus, regions worked, etc.

| **Sex** | Female | 4 |
| --- | --- | --- |
|  | Male | 9 |
| **Profession/ Job** | Researcher (Present) | 9 |
|  | User of biomedical technologies in Amazon communities (Past) | 5 |
| **Research Topic** | Infectious disease | 2 |
|  | Non-communicable disease | 1 |
|  | Diverse health topics | 8 |
|  | Developing health technologies | 2 |
| **Regions where they’ve worked** | Loreto, Ucayali, Madre de Dios, Junin, Cusco, San Martin |  |
| **Native from the Amazon** | No | 9 |
|  | Yes | 0 |
|  | No Answer | 4 |
| **Implemented biomedical technologies in Indigenous communities** | Yes | 10 |
|  | No | 3 |
| **Years of working in the Amazon, Years (SD)** |  | 11 (7.8) |

### Table 3 List of biomedical and general technologies used in the Peruvian Amazon region as stated by participants.

| **Biomedical Technologies** | | |  |
| --- | --- | --- | --- |
| **Prevention** | **Diagnosis** | **Treatment** | **General Technologies** |
| Sterilization Techniques  (e.g., UV lights, Aseptic cleaner) | Sphygmomanometer  (Blood Pressure Cuff)  *Hypertension* | Thermocoagulator  *Cervical Cancer* | Drones  *High resolution images to understand pathologies related to spatial analysis* |
| Tablet/ Mobile Devices  *Connection to health centres for emergency care (i.e., pregnancy)* | Glucometer and glycated hemoglobin test  *Diabetes* |  | Tablet/ Mobile Devices  *Data collection (via surveys, pictures, recordings) and communicating information to patients and community* |
| Vaccinations | Rapid Diagnostic Test  *HIV, STIs, ELISA, RPR, Malaria, Hepatitis B* |  | Computers  *Medical records* |
|  | Point of Care Device  *(e.g., LeadCare, for collecting blood lead level samples. Also used for algal viral infection diagnoses.)* |  | Cameras  *Data collection* |
|  | Hemoglobin Analyzer  *Anemia* |  | Digital Presentations  *Data dissemination* |
|  | Ultrasound Scanner |  | Fridge/ Cooler  *Regulate temperature of vaccinations and samples* |
|  | Centrifuge |  |  |
|  | Hemoglobinometer |  |  |
|  | Vital Testing Devices  *(e.g., Stethoscopes, Thermometers (including digital), Scales, Stadiometers)* |  |  |
|  | Serologic Test  *(e.g., IgM testing)* |  |  |
|  | Blood Lancets  *Blood sample collection* |  |  |
|  | Colposcope  *Cervical Cancer* |  |  |
| Telehealth (e.g., Video calls, Videoconferences, Tele-training) | | |  |

### Supplementary Information: Interview Questions

1. **Can you tell me about your roles and responsibilities in your job?**
   - What is your profession?
   - What is your topic of research and what department/unit are you in?
   - What is your role (e.g., what you do)?
   - What are your responsibilities (e.g., what you are accountable for or to do)?
   - Have you used, built, or designed biomedical technology over the past several years within your work? If so, please explain. (provide examples of biomedical technology)
2. **Can you tell me about the places and people that you work with in the Amazon?**
   - What region/district in the Amazon are you working with or have worked with (past and present)?
   - What Indigenous people (ethnicity) belongs to this community?
3. **Can you tell me about how much time you have spent in the Amazon over your career?**
   - How many years have you been working in the Amazon?
   - How often do you return to the Amazon (e.g., frequency – number of times and duration – length of visits)?
   - Are you a native or individual originating from the Amazon yourself?
4. **What health condition do you focus on?**
   - What technology do you currently use or have used for prevention, diagnosis, treatment, or investigating this health condition (e.g., specific names of technologies)?
   - What other technologies are used and for what use (e.g., blood pressure monitors, stethoscopes, etc.)?
   - What technology-based challenges do you face when using these mentioned technologies in the Amazon?
     - Using these technologies, would you agree or disagree that there a lack of resources or access because of the distances and remoteness? Why?
     - Do you believe these technologies sustainable? Why or why not? (e.g., Do they have short lifespans? Are they reusable?)
5. **In regard to physical environmental barriers, how do you find the implementation of these technologies into this specific climate, area, or environment?**
   - Does the humidity affect the efficiency of the technology? Provide examples.
   - Does remoteness affect that efficiency of the technology? Provide examples.
   - Does local infrastructure, available electricity, cost for maintenance, etc. effect the technology’s efficiency in either a positive or negative way? Can you provide specific examples?
6. **In regard to community barriers, what are some challenges faced? How do you find the cooperation and acceptability of the community with these technologies?**

- Who is using these technologies?
  - Are individuals (male or female) willing to learn how to use technologies? Why or why not?
  - Are these individuals trained? If so, is the training provided adequate or are there gaps? Please explain.
  - Is the technology being used properly? Are there any barriers hindering proper use?
- Who is more likely to cooperate and accept or allow the use of these technologies? Why?
- Who is more likely to not accept or not allow the use of these technologies? Why?
- How, if any, do factors such as cost, internet/electricity, language, gender, or culture influence the use of technologies?

1. **In regard to cultural barriers including community specific culture, personal culture, or any culture, what is your opinion on the effect of these technologies or challenges regarding the communities’ specific context – including traditions, beliefs, and exposure to technology?**
   - Based on your experiences, do any specific technologies, or technology in general, conflict with certain beliefs or values?
   - Based on your experiences, how do cultural traditions influence the use of these technologies?
   - Based on your experiences, does this affect a community’s willingness to participate?
2. **Based on your observations, what are positive and negative effects of technology on these communities?**
   - Are tradition and culture remaining intact? Why or why not? Provide specific examples.
   - Do the positive or negative effects outweigh each other? Do benefits outweigh barriers, or do barriers outweigh benefits? Please explain.
3. **Are there any issues with the design of this equipment in fitting the needs of this specific community?**
   - What specific design issues exist? Please explain and provide an example.
   - Have any interventions been used to overcome these design issues to meet community needs?
4. **For those who work in the design of these technologies: What are some of the biggest challenges faced that should be considered when deciding how it will be made or implemented?**

- Do these influence the effectiveness of the technology? Please explain.

1. **From your perspective, do you think producers or companies developing these technologies being used are taking context into consideration?**
   - How do you think context is or would influence the technology design?
   - Do you think there is value in adapting technology or implementing other forms that are community specific? Why?
2. **What are your thoughts on the use of mobile devices for collecting more frequent information regarding meteorological conditions (climate) and for telemedicine?**
   - Do you think the use of mobile devices to collect information on factors such as climate would be beneficial? Would this be acceptable in the current context? Please explain.
   - Do you think telemedicine (e.g., virtual connection to technology and health professionals), and reducing face-to- face contact, would have a positive or negative effect in implementing technology into communities? Please explain or provide examples where it would have a positive and negative effect.
   - What do you think about the training needs for the use of new technology within this cultural context?
3. **Would it be considered culturally appropriate and useful if health promotors had an app that could diagnose various diseases, e.g., malaria, anemia, and can report side effects back to Lima?**

- Would it overcome barriers? If so, please explain.

1. **That was my last question. Do you have any additional comments or items that you would like to share that we didn’t already cover today?**

### Table 4 Codebook descriptions used to define themes.

| **Theme** | **Coded Name** | **Code Description** |
| --- | --- | --- |
| **Primary** | **Design** | Development of technologies intended to accomplish goals in a particular environment, using a set of components, satisfying a set of requirements, and subject to constraints (31). In this study, it will involve the way technology is inspired (understanding one’s experience), ideated (how solutions are developed), and implemented in this environment (25). |
| **Primary** | **Implementation** | Implementation is defined as the process of putting a plan into effect (75). In this study, it will describe how technology is administered into communities. Additionally, implementation will be discussed in terms of perceptions of researchers that create these technologies and the considerations for implementation to proceed. |
| **Primary** | ***Acceptance, Acceptability, and Adoption*** | Technology *acceptance* in the context of this study involves perceptions of researchers after the use of technology (30).  Technology *acceptability* involves perceptions of technology before use of the technology, and will include perceived user satisfaction, positive feedback received, system usability, user engagement, and the actual use of the technology in a study or intervention (30).  Technology *adoption* is a multi-phase process beginning with the decision to adopt and then achieving continuous use (30). In this study, it is used to describe technologies that have already been implemented or successfully used by communities as perceived by participants. |
| **Secondary** | **Environmental Challenges** | Includes physical challenges that involve the environment including but not limited to challenges with temperature, terrain, weather, climate, and humidity. |
| **Secondary** | **Community Challenges** | Considers challenges surrounding the local rural community context such as level of institutional education and demographic. |
| **Secondary** | **Cultural Challenges** | Cultural challenges included but was not limited to beliefs, traditions, dress, religion, and language of the local community. |
| **Complementary**  **concepts** | **Challenge** | Challenge describes a situation faced by participants using technology in the Amazon that required greater effort to use the technology successfully. |
| **Complementary**  **concepts** | **Barrier** | Barrier describes an obstacle that prevented access to using the technology successfully. |
| **Complementary**  **concepts** | **Biomedical Technologies** | Technologies applied for the purpose of improving human health including medical equipment and health research equipment. |
